# Evaluation of elevated liver values in primary care - a series of studies on the status quo of care in Germany with special reference to alcoholic liver disease

**DOI:** 10.1186/s12875-022-01714-x

**Published:** 2022-05-03

**Authors:** Julian Wangler, Michael Jansky

**Affiliations:** Center for General Medicine and Geriatrics, University Medical Center of the Johannes Gutenberg University Mainz, Am Pulverturm 13, 55131 Mainz, Germany

**Keywords:** Liver, Transaminases, GP, Algorithm, Early detection

## Abstract

**Background:**

In primary care, elevated liver values often appear as incidental findings. As well considering the presenting symptoms, key factors in effective diagnosis are which liver values to include as indicators and when to refer patients for further diagnostics. It is also important that there is coordinated collaboration between GPs and specialists. There has hitherto been a lack of reliable findings on the status quo regarding the evaluation of (abnormally) elevated liver values in primary care.

**Methods:**

Between 2017 and 2021, four written explorative surveys of GPs and gastroenterological specialists were conducted in various German states, aimed at taking stock of the current status of GP-based diagnostics of (abnormally) elevated liver values. In addition, interviews were conducted with 14 GPs and gastroenterological specialists. This review article discusses the overall findings of the series of studies in a condensed manner at a higher level. The article aims to derive starting points for optimising the diagnosis of liver cirrhosis in primary care.

**Results:**

There are various challenges and problems associated with the evaluation of elevated liver values. For example, GPs draw on very different laboratory parameters, which are combined in different clusters. When elevated liver values are found, a majority of GPs prefer a controlled wait-and-see period, but often make use of direct referrals to specialists due to diagnostic uncertainties. GPs report interface problems with gastroenterological specialists, which are associated, among other things, with the preliminary evaluation that has been made and the timing of referral. Both GPs and specialists consider the introduction of an evidence-based diagnostic algorithm to be an important starting point for improving early detection and better coordination between healthcare levels.

**Conclusions:**

Efforts should be made to contribute to greater professionalisation and standardisation of primary care diagnostics and to better structure the interaction with gastroenterological specialists. These include a wider range of training formats, the development of a validated diagnostic pathway and the mandating of a liver function test as part of the check-up. The development of a GP-based guideline for managing elevated liver values also seems advisable.

**Supplementary Information:**

The online version contains supplementary material available at 10.1186/s12875-022-01714-x.

## Background

Elevated liver enzyme levels are a common incidental finding in primary care [1]. At the same time, the prevalence of elevated liver enzyme levels among patients receiving primary care is largely unknown [1, 2]. For the clinical area, studies such as the Gutenberg Heart Study showed that elevated liver values were found in around 20% of patients [3]. With regard to the prevalence in the general population, the SHIP study (cohort study of 4310 adults aged 20 to 79 years at baseline in Pomerania) showed an elevated ALT level in a quarter of all included patients [4]. According to estimates and projections, the prevalence of abnormal liver function tests (LFTs) depends on the definition and population but is likely to be between 15 and 20% in the general population [1].

Various studies have now demonstrated that elevated liver enzyme levels are associated with a higher rate of mortality and comorbidity [2, 5–10]. Common causes include alcohol abuse, use of medication, non-alcoholic fatty liver disease and viral infections [11–14]. Since elevated liver values can indicate life-threatening diseases, it is important that the healthcare system has the necessary prerequisites to detect and monitor elevated liver values at an early stage and, if necessary, to initiate further diagnostic steps.

In most cases, GPs are the first practitioners to discover (abnormally) elevated liver values in the course of a routine check-up [15–17]. In their role as primary care providers, they are responsible for assessing such findings and initiating further diagnostic steps. Given the time and resource constraints in the primary care setting, differential diagnostic workup for the early detection of liver disease can be challenging [12, 18–21]. Apart from possible warning signs (like hämatemesis, caput medusae etc.) and the question of which values, in which reference ranges and constellations, to include as meaningful indicators [17, 19, 22], the key element of a GP’s approach to (abnormally) elevated liver values is to differentiate between cases where a wait-and-see approach (with repetition of the laboratory tests) is advisable and where an immediate diagnosis is indicated, e.g. by direct referral to a specialist or to an outpatient liver clinic [17–19].

In Germany and other European countries, there are currently perceptible shortcomings in the consistent identification and evaluation of elevated liver values in the primary care setting [3, 15, 21–23]. On the one hand, a low proportion of early diagnoses is criticised, and on the other, an inconsistent differential diagnostic procedure that is highly dependent on the individual GP. One reason for this is thought to be the lack of structured, targeted screening programmes for chronic liver disease as part of the standard care regime [19, 20, 24]. In addition, there is a lack of an evidence-based and widely established diagnostic and clinical pathway to support GPs in the identification, classification and evaluation of elevated liver values, especially in the case of patients at high risk of developing cirrhosis [11, 19, 25–30]. In the case of Germany, there is also the fact that GPs have not yet been able to draw on explicitly GP-oriented, evidence-based guidelines.

To date, there are no systematic studies available in German-speaking countries that provide a reliable picture of the status quo of primary care in terms of elevated liver values and the associated challenges and problems. As a result, there is a lack of knowledge about GP procedures for dealing with (abnormally) elevated liver values, about the requirement for technical apparatus or possible interface problems between GPs and specialists.

### Overall study and research interest

This review summarises the results of a series of explorative studies and compares the results with existing research. The study, which consists of four sub-studies, stands as an independent supplementary study in the broader context of the Innovation Fund model project SEAL (Structured Early Assessment for Asymptomatic Liver Cirrhosis) for the early detection of liver fibrosis or asymptomatic liver cirrhosis.

The main research questions for the overall study were as follows:

- What are the prerequisites in the primary care setting for the detection and evaluation of elevated levels of liver enzymes?
- How do GPs classify and assess (abnormally) elevated liver values?
- How is the interdisciplinary collaboration between GPs and specialists in internal medicine or gastroenterology structured with regard to the diagnostic work up and the treatment of patients with elevated liver values?
- How could the early detection and diagnosis of liver disease in the primary care setting be improved (starting points for optimising the diagnosis of liver cirrhosis in primary care)?

## Methods

In line with the research questions, the aim was to take stock of the current status of GP diagnosis of (abnormally) elevated liver values. In particular, the aim should be to identify current practices, challenges and problems.

In order to gain the broadest possible knowledge relating to the formulated research interest, we sought the perspective not only of GPs but also registered doctors in gastroenterology practices.

Against the background of the findings, which are presented as a synopsis, the article aims to derive starting points for optimising the diagnosis of liver cirrhosis in primary care and making it more effective. The focus is therefore on weaknesses identified in the GP setting. All sub-studies were deliberately designed to be exploratory.

### Sub-studies

Based on a preliminary study in which 391 GPs in Rhineland-Palatinate were interviewed about their approach to elevated liver values [31], the questionnaire was updated for the purpose of more precise and in-depth investigations (among other things, supplementing item batteries on symptoms and diagnostics, prioritizing indicators for early detection of liver disease, questions on the frequency of liver disease in respective patients, use of guidelines and other recommendations for action), and the study was repeated on a significantly larger scale, in order to determine to what extent the first set of results are confirmed. This extended survey was conducted between October 2019 and March 2020 and gathered the views and experiences of a total of 2701 GPs in Hesse and Baden-Württemberg on their approach to elevated liver values [32].

An analogous procedure was followed for the survey of registered gastroenterology specialists. First, a preliminary survey was conducted in spring 2018, in which 54 gastroenterologists in Rhineland-Palatinate and Saarland were interviewed about their approach to elevated liver values and their collaboration with GPs [33]. The questionnaire, originally developed and conceptually tested by the authors in 2017, was updated and resulted in an extended study. Between April and October 2020, 313 doctors working in specialist gastroenterology practices in Baden-Württemberg, Hesse and Thuringia were interviewed in an online survey [34].

In addition, a smaller qualitative study was carried out after the aforementioned quantitative surveys. In the summer of 2021, seven GPs and seven established gastroenterological specialists in Rhineland-Palatinate (both randomly selected) were interviewed on the subject of clarification and interprofessional cooperation with regard to (unclear) elevated liver values.

Incentives were not used. Table 1 gives an overview of the studies described.

**Table 1:** Overview of the quantitative sub-studies carried out including information on socio-demographics

Group	General practitioners		Gastroenterological specialists	
Study	A [31]	B [32]	C [33]	D [34]
Study period	March–June 2017	October 2019–March 2020	January–March 2018	April–October 2020
N (response rate)	391 (16%)	2701 (26%)	54 (40%)	313 (59%)
Gender	60% male, 40% female	61% male, 39% female	83% male, 17% female	84% male, 16% female
(Specialist) Background	100% General Practice	75% General Practice, 25% Internal medicine (working as GP)	67% specialists in internal medicine and gastroenterology, 29% specialists in internal medicine, 4% other	65% specialists in internal medicine and gastroenterology, 28% specialists in internal medicine, 7% other
Mean age	51 (Median: 52)	52 (Median: 53)	54 (Median: 55)	58 (Median: 57)
Office setting	47% in medium-sized and large towns or cities, 53% in small towns or rural areas	49% in medium-sized and large towns or cities, 51% in small towns or rural areas	80% in medium-sized and large towns or cities, 20% in small towns or rural areas	67% in medium-sized and large towns or cities, 33% in small towns or rural areas
Type of office	49% individual doctor’s offices, 48% joint offices, 3% other	51% individual doctor’s offices, 46% joint offices, 3% other	33% individual doctor’s offices, 63% joint offices, 4% other	40% individual doctor’s offices, 57% joint offices, 3% other
Patients per quarter	23% < 1.000, 37% 1.000–1.500, 19% 1.501–2.000, 21% > 2.000	19% < 1.000, 38% 1.000–1.500, 19% 1.501–2.000, 24% > 2.000	25% < 1000, 23% 1000–1500, 25% 1500–2000, 27% > 2000	30% < 1000, 19% 1000–1500, 22% 1500–2000, 29% > 2000

### Development of survey instruments

Since the studies built on each other, there was a continuous learning process which informed the design of the subsequent sub-study. In addition, the survey instruments developed were supported by other elements:

- Preparations and exchanges within the SEAL project
- GP survey: The questionnaire [31], originally developed in 2017, was enriched by a group discussion with 10 GPs during the development process.
- Specialist survey: Several experts from the Cirrhosis Centre of University Hospital Mainz were consulted during the development process, in order to check the completeness and appropriateness of the questionnaire [33] from a specialist’s point of view and to align the questionnaire closely with the reality of care.
- Other preliminary studies by the authors on structured, evidence-based primary care ([35] inter alia)
- General literature searches in the design of all sub-studies (papers focusing on the assessment of elevated liver enzymes in primary care were used here) ([36] inter alia)
- Carrying out pre-tests in the run-up to data collection

The aim was to keep the instruments used to interview GPs and gastroenterologists mutually compatible. To this end, certain questions in both questionnaires (e.g. attitude to the wait-and-see approach, starting points for optimising early detection) were worded in a very similar way.

The survey instruments for the two more comprehensive surveys are included in the Additional file 1.

### Data analysis

Data from the quantitative studies were evaluated using SPSS 23.0. In order to highlight different approaches adopted by GPs, in addition to descriptive analysis, the method of factor analysis (Varimax rotation) was used, in which variables are combined into factors on the basis of systematic relationships (correlations). After data collection, the team evaluated the resulting transcripts using qualitative content analysis according to Mayring [36]. Our focus lay on forming logical categories from the various opinions and experiences. Selected citations are presented to support the quantitative findings.

## Results

**Fig. 1:**
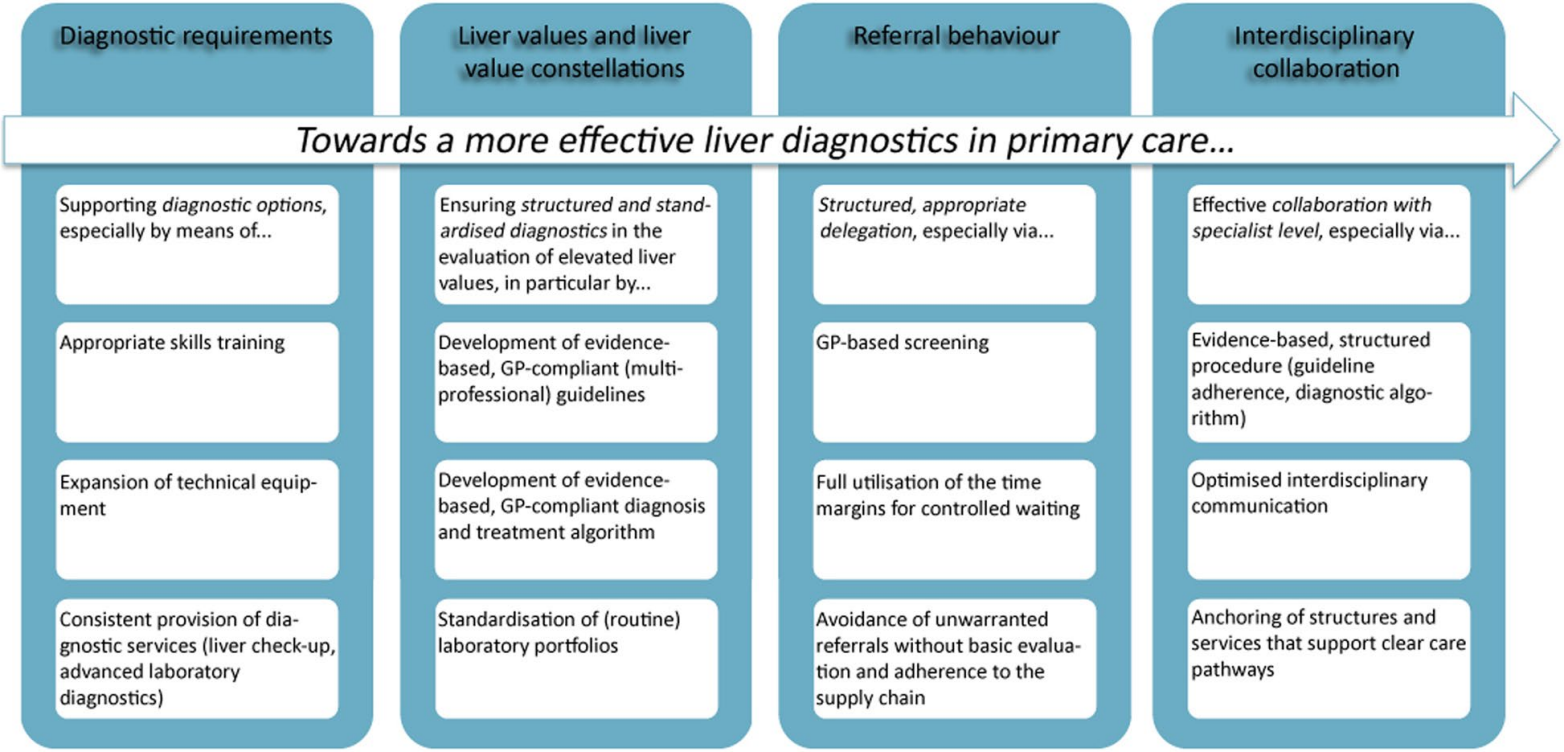
Derived starting points for effective liver diagnostics by GPs (own figure)

Figure 1 shows the starting points condensed from analysis of the sub-studies with a view to more effective liver diagnostics by GPs. In the following, each of the dimensions presented will be discussed with reference to the respective central findings and correlated with existing research. The surveys with the much larger samples serve as the primary reference [32, 34].

### Liver values and liver value constellations

The GP survey [32] has shown that, in everyday practice, there is a strong focus on a comparatively small number of selected liver parameters in the evaluation of (abnormally) elevated liver values. For example, γ-GT is the main laboratory value considered (95%). About two-thirds (65%) include aspartate aminotransferase (ASAT, AST, GOT) in their analysis, followed by alanine aminotransferase (ALAT, ALT, GPT) (63%), alkaline phosphatase (AP) (62%) and platelet count (57%). In a questionnaire, the respondents were asked to name which three indicators they considered to be most important and meaningful for the early detection of liver cirrhosis. Analogously, γ-GT (92%), aspartate aminotransferase (83%) and alanine aminotransferase (79%) are mentioned here, while other values lag a considerable distance behind.

At the same time, a factor analysis revealed a strongly heterogeneous and divergent approach on the part of GPs in the diagnosis of potential chronic hepatic parenchymal disease (see Table 2). Thus, GPs not only pay attention to very different symptoms, but also use different liver-associated laboratory parameters or value constellations as indicators for the identification of (incipient) liver disease within the framework of laboratory diagnostics ordered by GPs. While one cluster focuses on functional parameters such as bilirubin, PT according to Quick (INR), cholinesterase and albumin, another primarily looks at indicators of toxic cell damage or liver disease that has already occurred. Among other parameters, alanine aminotransferase receives particular attention. In addition, a third cluster that focuses on γ-GT as a parameter for possible liver disease stands out.

*“We general practitioners are all-rounders in our day-to-day practice. The classification of liver values and the consideration of limit values is a very specific profession. Especially with moderate increases, it is not always easy to draw the right conclusions. I admit it's hard for me.”* (Interviewed GP 3m)

The survey of registered gastroenterologists [34] was able to confirm that, from the specialist’s perspective, GPs rely on highly divergent liver values to guide their everyday practice. For example, 57% of specialists experience it as a considerable inconvenience to have to constantly readjust to the diagnostic requirements due to the difference and lack of standardisation in the collection of liver values on the part of GPs. The spectrum for conclusions and further care decisions is correspondingly diverse.

*“If you ask me: The behavior of general practitioners when considering and interpreting liver values is too uncontrolled and not supported enough by evidence-based guidelines. As a result, we often have to adapt our work to the preparatory work done by GPs from scratch.”* (Interviewed gastroenterologist 1m)

**Table 2:** Laboratory values observed. Question: Which laboratory findings potentially linked to liver disease do you usually examine in routine lab work for general screening check-ups? (*N* = 2.701, GPs)

		Rotated component matrix		
	Overall agreement	Comp. 1 (Expl. variation: 26,1%)	Comp. 2 (Expl. variation: 18,2%)	Comp. 3 (Expl. variation: 10,6%)
Alanine aminotransferase (ALAT, ALT, GPT)	63%	−.055	.774	−.119
γ-glutamyltransferase (GGT)	95%	−.040	−.018	.884
Aspartate aminotransferase (ASAT, AST, GOT)	65%	.141	.576	.386
AP (alkaline phosphatase)	62%	.542	.252	.305
Ferritin	26%	.734	.035	−.102
Bilirubin	46%	.686	.219	.213
PT according to Quick (INR)	27%	.663	.207	−.165
Cholinesterase	19%	.675	.139	−.023
Albumin	23%	.740	−.027	.092
Platelet count	57%	.256	.715	−.112
MCV	55%	.192	.614	.165

Extraction method: Principal component analysis

Rotation method: Varimax, Kaiser normalisation

Rotation in 4 iterations for convergence

Total explained variation: 54.9%

Sampling adequacy, Kaiser-Meyer-Olkin: .787

Significance, Bartlett: *p* < 0.001

### Diagnostic requirements

The GP survey [32] showed that 29% of the GPs included in the study offer a special liver check-up in their own practice in addition to the SHI screening. On the other hand, 66% do not offer such a service to supplement the SHI health check-up. In terms of the prerequisites for diagnostic equipment, standard upper abdominal sonography for the identification and further evaluation of liver disease is usually available in most GP practices (89%), and more rarely extended laboratory diagnostics (64%).^1^ 5% respectively offer an elastography or fibroscan investigation.

As was found in a detailed interrogation via item set, the surveyed GPs focus on certain indicators of incipient liver disease that prompt more in-depth diagnostics, while paying less attention to other indicators. From their previous experience, GPs pay particular attention to excessive alcohol consumption (94%) and also to signs such as upper abdominal complaints (76%), symptoms of fatigue (75%), ascites (71%), itching (71%) and skin changes (65%). In the experience of those surveyed, symptoms such as loss of appetite, weight loss, Dupuytren’s contracture, or gynaecomastia are less often a sign of potential liver disease.

Associated with this, there is evidence that GPs experience a lack of diagnostic certainty and a lack of guidance options when clarifying (abnormally) elevated liver values. For example, 38% consider themselves to be very or quite competent in the evaluation of elevated liver values, while around 50% consider themselves to be less or not at all competent in this area. Only one third of the surveyed GPs have consulted practice or action recommendations, expert opinions of medical societies or diagnostic pathways offered by healthcare providers (e.g. German Liver Foundation). The interest articulated by a majority of respondents in an expansion of adequate further training offerings is also an indication of the need for the training of GPs in this area.

*“In my opinion, there should be more evidence-based tools tailored to general practitioners – on this topic in particular.”* (Interviewed GP 5w)

From the point of view of the specialists interviewed [34], it would also be useful if there were more training formats that gave GPs more confidence in evaluating liver values, as this would have a direct impact on the quality and effectiveness of interdisciplinary collaboration.

*“Fromt my point of view, primary care could do better at initial testing and diagnosis of (incipient) liver disease.”* (Interviewed gastroenterologist 4m)

### Referral behaviour

In the light of the study results, the referral behaviour of GPs reveals identifiable inconsistencies. On the one hand, almost two-thirds of the GPs surveyed [32] consider it sensible to initially practice a wait-and-see approach of several weeks (median: 5.0) after detecting moderately elevated liver values, and therefore only to consider referral to a higher specialist level after a repeat investigation at a later point in time. However, the respondents give divergent information about their actual referral behaviour, which they justify, in an open question, primarily on the basis of diagnostic uncertainties. Thus, around 40% state that they usually refer patients directly to a specialist or even to a specialist outpatient clinic after noticing abnormally elevated liver values. Only 32%, on the other hand, have consistently waited.

*“Of course, the manual says ‘controlled waiting’. But the reality is sometimes different. Elevated liver enzymes are complex and to be honest I sometimes feel overwhelmed on the subject. That's why I tend to transfer as quickly as possible.”* (Interviewed GP 2w)

79% of the GPs reported that they had referred their patients to a gastroenterology practice; 44% had referred them directly to a liver outpatient clinic and 27% to a gastroenterology department or clinic.

The results attest to the central pilot role of the GP within the healthcare system. 98% of the internal medicine specialists surveyed stated that patients with (abnormally) elevated liver values are usually referred by their GP. 23% mention referral by another specialist and 20% that patients visit their practice on the advice of the clinic (40% self-referrals by the patient).

From the perspective of gastroenterological specialists [34], it can be seen that they, for their part, criticise the referral behaviour of GPs, which, in their opinion, is often either significantly premature (64%) or too tardy (57%). In addition, patients with slightly or moderately elevated liver values often turned out to be not affected by (incipient) liver disease (69%).

*“In my experience, many general practitioners are even too quick to refer patients with elevated liver values of unknown aetiology. […] For example, many simply look at γ-GT and refer patients to specialists for small increases. […] It would be good if they could do part of the diagnostic work themselves and thus better pre-select our patients.”* (Interviewed gastroenterologist 4m)

### Interdisciplinary collaboration

Coordinated collaboration between GPs and specialists is essential for an effective, early diagnosis to explain elevated liver values and initiate appropriate treatment. Although both GPs and specialists experience collaboration with the other side as positive in the majority of cases, considerable interface problems and hurdles in interdisciplinary interaction are articulated.

Apart from a lack of specialised internal medicine practices in the vicinity (73%), frequent difficulties for GPs [32] are a lack of accessibility to discuss the usually complex patient problems (69%) (see Table 3). 90% state that there are often longer waiting times for an appointment for differential diagnostic assessment for suspected liver disease. In rural areas, these challenges are exacerbated due to the significantly lower density of specialists. Another considerable problem experienced by GPs is that patients are not sufficiently informed about their condition by their specialist colleagues and so return to the GP due to uncertainty (72%). Likewise, the referral behaviour of specialists following the diagnosis of liver disease seems to be characterised by frequent referrals back to the GP (63%). In the absence of prompt presentation to a specialist outpatient clinic, there is at least a risk of the patient entering an unnecessary loop as a result of being referred back.

*“Working together with specialist colleagues is full of difficulties and stumbling blocks. Maybe it has something to do with the fact that in Germany the sectors are too separated from each other, but I can't say that I'm usually well informed about what diagnostic steps the gastroenterological colleague takes. Or what I have to do when the patient comes back to me.”* (Interviewed GP 4m)

**Table 3:** Challenges experienced in the interdisciplinary relationship, GPs. Question: A variety of challenges may arise when general practitioners and district specialists for outpatients collaborate on diagnosing cirrhosis. How often have you experienced the following challenges? (*N* = 2.701, GPs)

Statement	Frequently	Occasionally	Rarely	Never	No response
*Resident gastroenterologists are fully booked long-term due to the many gastroduodenoscopy and colonoscopy tests they are required to perform.*	69%	21%	6%	3%	1%
*District specialists do not have the time to discuss mostly complex patient problems with you.*	41%	39%	10%	8%	2%
*There are too few nearby specialist internal medicine practices to diagnose liver counts the way I would like.*	37%	36%	11%	15%	1%
*Specialists do not brief patients enough, who then go back to general practitioners out of uncertainty.*	30%	42%	13%	13%	2%
*Gastroenterological district specialists are difficult for patients to reach.*	35%	34%	16%	14%	1%
*Specialists do not issue direct referrals to a liver centre on suspicion of cirrhosis, so patients come back to their general practitioners for the time being (going around in circles with time wasted).*	23%	40%	20%	16%	1%
*District specialists are booked out for too long, so I refer my patients straight to a specialist clinic.*	21%	35%	19%	23%	2%
*I have to wait for a long time for district specialists to pass on their findings.*	19%	33%	20%	27%	1%
*District specialists do not inform general practitioners enough about the tests they have conducted or the results and/or diagnoses they have made.*	17%	35%	24%	23%	1%

The views expressed by gastroenterological specialists [34] show that they are also critical of the interaction with primary care (see Table 4). Apart from the timing of patient presentation, the GP’s decision not to make a genuine basic assessment and to refer based on suspicion or doubt is experienced as a significant problem in the interaction with GPs (71%). From the point of view of registered gastroenterologists, further impediments to the interaction with GPs arise from the fact that the latter do not always follow up on elevated liver values (65%). Some of the respondents see the fact that GPs often follow a very different procedure for evaluating elevated liver values (e.g. collection of different liver values, 57%) as an additional hurdle and this corresponds with the impression articulated by specialists that the investigations, results and diagnoses are not always transparent (63%). As a result of such interdisciplinary problems, 84% of the specialists report that they frequently (25%) or occasionally (59%) encounter patients with liver disease that has not been detected by the GP.

*“I don't think the general practitioners or the specialists are at fault. Communication and patient treatment simply need to be better interlinked between the different levels of care. It would need a mechanism that creates a certain uniformity. I could well imagine an established and well-tested algorithm here.”* (Interviewed gastroenterologist 1m)

**Table 4:** Challenges experienced in the interdisciplinary relationship, gastroenterological specialists. Question: A variety of challenges may arise when gastroenterologists and general practitioners work together to diagnose and treat cirrhosis. How often have you experienced the following challenges? (*N* = 313, gastroenterological specialists)

Statement	Frequently	Occasionally	Rarely	Never	No response
*I have detected (incipient) liver disease that the general practitioner did not notice or remained unaware of in a patient.*	25%	59%	11%	4%	1%
*Primary care could do better at initial testing and diagnosis of (incipient) liver disease.*	29%	42%	17%	10%	2%
*General practitioners are not always sufficiently aware of elevated liver values with unknown aetiology to notice the onset of liver disease at an early stage.*	27%	43%	13%	13%	4%
*Patients that general practitioners have referred to gastroenterologists for an elevated liver count of unknown aetiology often turn out to be non-specific.*	18%	51%	15%	12%	4%
*General practitioners often fail to follow up on elevated liver values.*	23%	42%	17%	15%	3%
*General practitioners are too quick to refer patients with elevated liver values of unknown aetiology to gastroenterologists, leaving gastroenterologists booked out for long periods of time.*	34%	30%	19%	14%	3%
*General practitioners do not adequately inform gastroenterologists about the tests they perform, the results and/or the diagnoses they have made.*	20%	43%	20%	16%	1%
*General practitioners are inconsistent in their approach to analysing liver values; this may include varying liver values recorded depending on the general practitioner, so specialists need to keep adjusting to the preliminary work performed by general practitioners.*	35%	22%	22%	20%	1%
*General practitioners wait too long before referring patients with an elevated liver count of unknown aetiology to a gastroenterologist.*	30%	27%	25%	16%	2%

### Approaches for optimising primary care

Respondents were given a list of various potential measures to increase the proportion of patients diagnosed early. There is a high level of agreement between the GPs and specialists included in the study. In view of the perceived inconsistency in the approach to evaluating elevated liver values in the outpatient sector, as well as existing interface problems, 80% of GPs and 85% of specialists support the introduction of a structured, evidence-based and broadly applicable diagnosis and treatment algorithm as a (highly) effective measure. 65% of GPs and 55% of specialists see an expansion of the laboratory workup included in the health check-up from the age of 35 as an effective measure. 61% of GPs and 60% of specialists consider the development of an explicit, evidence-based S3 guideline for the systematic evaluation of elevated liver values to be particularly effective. 50% of GPs and 52% of specialists are in favour of introducing a genuine liver check as part of the SHI regime.

In addition, 70% of GPs and 76% of specialists believe that a significant expansion of various kinds of training events for GPs on how to evaluate liver enzyme levels and practice a structured interaction within the healthcare chain would be (very) effective.

## Discussion

### Principal findings and comparison with prior work

The wide-ranging survey of GPs and specialists in internal medicine and gastroenterology in several large German states [32, 34] confirms that elevated liver enzymes levels are a common finding in primary care. This is associated with the need for systematic and consistent evaluation as well as functioning collaboration with higher healthcare levels. The survey results confirm the findings of the previous studies [31, 33] in all areas and indicate that there are a number of weaknesses in the management of elevated liver values in primary care.

- 1) There is a strongly heterogeneous and divergent approach on the part of GPs in the evaluation of potential chronic hepatic parenchymal disease. For example, GPs not only pay attention to very different symptoms, but also use different liver-associated laboratory parameters or value constellations as indicators for the identification of (incipient) liver disease within the framework of laboratory diagnostics ordered by GPs. Three clusters were identified in the analysis of laboratory values. These are probably related to the fact that the laboratories used do not provide identical portfolios. At the same time, there seems to be a tendency among some GPs to focus on as few easy-to-grasp parameters as possible in their everyday practice [19, 29]. Especially the level of γ-GT seems to be an obvious and often exclusive indicator for many GPs, although an elevated γ-GT level on its own, in the absence of alcohol consumption, does not necessarily indicate liver pathology [21, 37, 38].
- 2) GP practices do not always have the necessary diagnostic prerequisites for an adequate assessment of elevated liver values. This applies to technical equipment as well as the consistent availability of diagnostic services (e.g. liver check-up). GPs themselves articulate a distinct need for training in diagnostic skills and/or in the expansion of adequate advanced education offerings in this area and this is reflected, inter alia, in the fact that indicators of (incipient) liver disease are often selectively recorded. Both in terms of medical history and test results, GPs seem to focus more on lifestyle-related liver diseases such as alcoholic liver disease and less on the hepatological problem of fatty liver, viral liver diseases and systemic autoimmune phenomena [32]. As well as the GPs’ self-assessment determined in the course of the survey, the fact that practice recommendations and expert opinions of medical societies are used somewhat rarely also speaks in favour of greater support for the diagnostic skills of GPs.
- 3) There is evidence that GP referral behaviour is not always appropriate when it comes to the need for assessment of (abnormally) elevated liver values. This relates in particular to the extent to which a controlled wait-and-see period is required after the detection of elevated liver values and at what point a referral is indicated. Although, in principle, the majority of respondents prefer a controlled waiting period of up to 8 weeks, they make use of faster and more frequent referrals to specialists or liver outpatient clinics in everyday practice. Diagnostic uncertainties as well as system-related limitations (time required for in-depth analysis, laboratory budgeting, etc.) may also be partly responsible for this. According to the specialists interviewed, it is more common for GP referrals to be made too early or too late. Besides, GPs are not always sure what would be suitable lab requests for their respective patient [35].
- 4) There are various challenges in the interdisciplinary interaction and communication between GPs and registered internal medicine/gastroenterology specialists. Especially problematic for GPs are the lack of accessibility and the fact that there are quite often long waiting times for an appointment for differential diagnosis in the case of suspected liver disease. Moreover, the referral behaviour of specialists following the diagnosis of liver disease seems to be characterised by frequent referrals back to the GP [16, 17, 21]. However, the survey of gastroenterological specialists shows that the GP’s failure to provide a genuine basic assessment can be a significant problem in the interaction with specialists. According to some of the respondents, the fact that GPs often have a very divergent approach to the evaluation of elevated liver values is an additional hurdle.

The findings and problems that were identified can be summarised as follows: the management of elevated liver values found in the course of a general blood test is a diagnostic challenge, which has hitherto been highly dependent on the individual approach of each GP, so that corresponding actions have been very heterogeneous. The results might correlate to the absence of a validated, widely accepted diagnostic algorithm for the identification of patients with elevated liver enzymes at high risk for liver cirrhosis in primary care [27–30]. Such a structured diagnosis and clinical pathway applied right across the healthcare system could be a valuable tool for evidence-based professionalisation and standardisation of GP practice [24, 26, 28].

For some time now, various research and support networks as well as professional societies have been pointing out the importance of a systematic diagnostic pathway. In connection with this, they have developed algorithms that can be adequately applied when elevated liver values are found [39]. Proposals have already been made for how a systematic and practicable diagnostic procedure could be structured for such a clinical pathway [12, 40, 41]. Holstege categorises procedures into three different groups based on the pattern of pathologically altered liver values [42]. If transaminases are elevated, it should first be clarified whether there is a viral genesis, a genetic metabolic disease or drug-related toxic damage. Where cholestasis enzyme levels are elevated, sonography should be used to determine whether the cause of cholestasis is intra- or extrahepatic. And last but not least, the generation and widespread establishment of a practical, situational algorithm for (further) evaluation of elevated liver values would be valuable in overcoming interface problems [18, 37]. This would lead to better structuring of the differential diagnostic procedure, avoid hasty or late referrals, optimise the information flow and ensure a smoother division of labour between GPs and specialists [34].

Four out of five GPs are in favour of the introduction of a structured diagnostic algorithm and do not see this as interfering with their therapeutic freedom [32]. The same proportion of specialists advocate the introduction of a structured diagnostic algorithm [34]. However, an important prerequisite for the successful introduction of such an instrument will be that it is oriented as closely as possible to the reality of primary care [16, 37]. This includes, among other things, the influence of costs and time expenditure, which must be taken into account by an evidence-based diagnostic path with regard to questions of clarification and referral behaviour [17].

International studies suggests that a robust diagnostic algorithm applied right across the healthcare system could generate key benefits, including cost-benefit effects, more consistent adherence to the chain of care, quicker early detection, more effective follow-up, and more individually tailored treatment that can prevent disease progression and even lead to cirrhosis regression [26–30]. These could be combined with additional measures, structures and services that support clear care pathways, such as targeted training formats, firm anchoring of liver enzyme-associated blood tests within the framework of the GP check-up, standardised parameters for routine laboratory tests and the development of an evidence-based, GP-oriented guideline for the detection and management of elevated liver enzyme levels [15–17, 29, 37].

### Strengths and limitations

The surveys that are presented here had already been conceptually tested on the basis of several preliminary studies and were tailored to GP and specialist care provision. In the course of the implementation, it was possible to obtain large, mixed samples, which provide a broad picture of GPs’ approaches to evaluating liver enzyme levels, as well as the corresponding prerequisites. That said, none of the presented studies can claim to be representative (e.g. regional focus, limited response). Furthermore, due to anonymisation - which was a prerequisite for broad participation - it is not possible trace from which parts of the respective federal states GPs or specialists participated. Equally, it is possible that doctors with a greater interest in the subject have been more willing to participate.

Notable limitations, especially relating to the GP survey, are that, both in terms of medical history and results, greater emphasis is placed on alcoholic liver disease and less on the problems of fatty liver, viral liver diseases and systemic autoimmune phenomena [43]. Thus, the survey cannot comprehensively address the full spectrum of liver disease in primary care. Follow-up studies will be required to address this gap in the research.

It would be interesting for future studies to identify which measures GPs with hepatological expertise think have given them greater confidence in the management of elevated liver values and which measures these clinicians think should be taken to improve the effectiveness of early detection in primary care.

## Conclusions

Elevated liver enzyme levels are a common incidental finding in primary care. It is therefore all the more important to carry out effective assessment and exclusion diagnostics in order to avoid any existing liver disease being overlooked. For this, it is not only relevant which liver values are used, in which constellations or when patients are referred for further investigation but the quality of a functioning interaction between GPs and specialists is also crucial.

The study results indicate that currently early, consistent identification and evaluation of (abnormally) elevated liver values are not always possible in the primary care setting due to various barriers and challenges. In order to successively increase the effectiveness of primary medical care, it seems advisable to take measures that contribute to greater professionalisation and standardisation of diagnostics and to structure the interaction with gastroenterological specialists more effectively. In this context, the establishment of a sufficiently validated diagnosis and clinical pathway oriented to the reality of outpatient care can be a valuable instrument. It would also make sense to offer a broader range of topic-related training and further education formats and to include blood tests for liver enzyme levels as a mandatory component of medical check-ups. The development of an evidence-based GP-oriented guideline for the detection and management of elevated liver values seems advisable in order to provide GPs in the outpatient sector with better and tailored guidance for the diagnostic assessment of liver enzyme levels. With the support of the above-mentioned measures, GPs should be more confident in carrying out basic diagnosis of abnormally elevated liver values themselves and then referring patients as necessary, thus fulfilling their referral role in an optimum manner.

## Supplementary Information

**Additional file 1: Appendix 1.** Survey of general practitioners [32]. **Appendix 2.** Survey of gastroenterologists [34].

## Data Availability

All major data generated or analysed during this study are included in this published article. Additional information can be provided on request made to the corresponding author.

## Abbreviation

GP(s): General Practitioner(s)

## Footnotes

1 During the extended laboratory examination, liver and kidney values plus electrolytes are determined in addition to the blood count. This extended laboratory check is usually billed privately as a so-called private service.
