# Supplementary material for: Evaluation of elevated liver values in primary care - a series of studies on the status quo of care in Germany with special reference to alcoholic liver disease
Source: BMC Prim Care. 2022 May 3;23:104. doi: 10.1186/s12875-022-01714-x (PMC9063320; doi:10.1186/s12875-022-01714-x)
Supplement: Supplementary file 1 — Additional file 1: Appendix 1. Survey of general practitioners [32]. Appendix 2. Survey of gastroenterologists [34]. [file 12875_2022_1714_MOESM1_ESM.zip › questionnaire_GP survey_translation.docx]

**1. Does your medical practice provide separate liver check-ups alongside the usual statutory health insurance health check-ups?**

⃝ Yes, in addition to statutory health insurance health check-ups ⃝ Yes, in a separate liver check-up ⃝ No

**2. Which of the following general and follow-up diagnostic tests for liver diseases does your medical practice provide?**

⃝ Upper abdominal sonography ⃝ Extended laboratory diagnostics ⃝ Other:

**3. In general: What do you see as the most frequent indications of incipient liver disease, and what would prompt you to make more in-depth follow-up diagnostics?**

⃝ Long history of alcohol consumption ⃝ Upper abdominal complaints ⃝ Bowel movement and digestion problems

⃝ Tiredness, listlessness ⃝ Recurrent heart problems ⃝ Suspected alcohol abuse

⃝ Persistent diarrhoea ⃝ Chronic pruritus ⃝ Recurrent bladder infection

⃝ Recurrent nosebleed ⃝ Persistent headache ⃝ Loss of appetite

⃝ Gynaecomastia ⃝ Ascites ⃝ Characteristic skin alterations (spider naevi etc.)

⃝ Carpal tunnel syndrome ⃝ Multiple bruising ⃝ Genital mycosis

⃝ Dupuytren’s contractures ⃝ Changes in bodyweight

Other:

**4. Which laboratory findings potentially linked to liver disease do you usually examine in routine lab work for general screening check-ups?**

⃝ Alanine aminotransferase (ALAT, ALT, GPT) ⃝ GGT (Gamma-GT) ⃝ Aspartate aminotransferase (ASAT, AST, GOT)

⃝ AP (alkaline phosphatase) ⃝ Ferritin ⃝ Bilirubin

⃝ Quick (INR) ⃝ Cholinesterase ⃝ Albumin

⃝ Platelet count ⃝ MCV

Other:

**5. If you were forced to decide: What in your opinion would be the** **three most important** **indicators in early diagnosis of cirrhosis?** (Up to three responses)

⃝ Alanine aminotransferase (ALAT, ALT, GPT) ⃝ GGT (Gamma-GT) ⃝ Aspartate aminotransferase (ASAT, AST, GOT)

⃝ AP (alkaline phosphatase) ⃝ Ferritin ⃝ Bilirubin

⃝ Quick (INR) ⃝ Cholinesterase ⃝ Albumin

⃝ Platelet count ⃝ MCV

Other:

**6. Please give an estimate: What percentage of patients at your medical practice have an elevated liver count?** (not how many patients you have diagnosed with elevated liver enzymes, but your general estimate of elevated liver enzyme prevalence amongst your patients as a whole.)

Around % ⃝ Can’t say, no answer

**7. From your own experience, what do you think: What percentage of patients with an elevated liver count at your practice have also had clinical symptoms?**

Around  **___** % ⃝ Can’t say, no answer

**8. How often do you follow medical association recommendations such as practice recommendations, guidelines, algorithms while diagnosing elevated liver values?**

⃝ Frequently ⃝ Occasionally ⃝ Rarely ⃝ Never ⃝ Don’t know

**9. General practitioners have varying opinions on how to deal with a moderately elevated liver count. Some think it is better to wait after diagnosing a moderately elevated liver count and check them again at a later follow-up. Others prefer direct referral to a specialist physician or clinic. Leaving special cases or unambiguous individual cases aside, what do you think makes more sense?**

⃝ Wait and watch ⃝ Referral to a specialist ⃝ Referral to a specialist clinic

⃝ Difficult to say, undecided ⃝ No answer

**10. Assuming you decide to wait for now and check again at a later follow-up: How long do you think you should wait for the follow-up?**

weeks

**11. Thinking about the patients you have diagnosed with an elevated liver count of unknown aetiology or explanation in the past few years: Have you usually referred them straight to a specialist physician or clinic or used the wait-and-watch strategy instead?**

⃝ Usually referred them ⃝ Usually used the wait-and-watch approach

⃝ Combination ⃝ No answer

*Only answer the following question if you answered 'usually referred' or 'Combination,' otherwise go straight to question 13.*

**12. Where did you refer your patients to?**

⃝ Gastroenterological clinic ⃝ Gastroenterological hospital or department of a hospital

⃝ Specialist liver clinic ⃝ Haematological clinic

⃝ Specialist haematological clinic or department in a hospital ⃝ No answer

**13. Cirrhosis is usually diagnosed at an advanced stage because symptoms are usually relatively non-specific at first. How effective do you think the following measures would be in increasing the number of patients diagnosed early?**

Introduction of a separate liver check in statutory health insurance
⃝ Very effective ⃝ Rather effective ⃝ Not or not very effective ⃝ Don’t know

Extension of lab testing in health check-ups for patients aged 35 and above
⃝ Very effective ⃝ Rather effective ⃝ Not or not very effective ⃝ Don’t know

Establishment of a structured diagnosis and therapy algorithm with specific instructions for general practitioners on dealing with an elevated liver count in patients
⃝ Very effective ⃝ Rather effective ⃝ Not or not very effective ⃝ Don’t know

Increased training for general practitioners to improve early detection of cirrhosis
⃝ Very effective ⃝ Rather effective ⃝ Not or not very effective ⃝ Don’t know

Development of an evidence-based GP-appropriate guideline (S3) for methodical diagnosis of elevated liver values, possibly of unknown aetiology (such as DEGAM)
⃝ Very effective ⃝ Rather effective ⃝ Not or not very effective ⃝ Don’t know

**14. From your own experience, how do you rate collaboration between general practitioners and district specialists in diagnosing elevated liver enzyme levels of unknown aetiology or diagnosing cirrhosis?**

⃝ Very good ⃝ Rather good ⃝ Rather poor ⃝ Very poor ⃝ Difficult to say ⃝ No answer

**15. Also from your own experience, how do you rate collaboration between general practitioners and specialist clinics in diagnosing elevated liver enzyme levels of unknown aetiology or diagnosing cirrhosis?**

⃝ Very good ⃝ Rather good ⃝ Rather poor ⃝ Very poor ⃝ Difficult to say ⃝ No answer

**16. A variety of challenges may arise when general practitioners and district specialists for outpatients collaborate on diagnosing cirrhosis. How often have you experienced the following challenges?**

Gastroenterological district specialists are difficult for patients to reach.
⃝ Frequently ⃝ Occasionally ⃝ Rarely ⃝ Never

District specialists do not have the time to discuss mostly complex patient problems with you.
⃝ Frequently ⃝ Occasionally ⃝ Rarely ⃝ Never

Resident gastroenterologists are fully booked long-term due to the many gastroduodenoscopy and colonoscopy tests they are required to perform.
⃝ Frequently ⃝ Occasionally ⃝ Rarely ⃝ Never

I have to wait for a long time for district specialists to pass on their findings.
⃝ Frequently ⃝ Occasionally ⃝ Rarely ⃝ Never

District specialists do not inform general practitioners enough about the tests they have conducted or the results and/or diagnoses they have made.
⃝ Frequently ⃝ Occasionally ⃝ Rarely ⃝ Never

District specialists are booked out for too long, so I refer my patients straight to a specialist clinic.

⃝ Frequently ⃝ Occasionally ⃝ Rarely ⃝ Never

Specialists do not issue direct referrals to a liver centre on suspicion of cirrhosis, so patients come back to their general practitioners for the time being (going around in circles with time wasted).
⃝ Frequently ⃝ Occasionally ⃝ Rarely ⃝ Never

Specialists do not brief patients enough, who then go back to general practitioners out of uncertainty.
⃝ Frequently ⃝ Occasionally ⃝ Rarely ⃝ Never

There are too few nearby specialist internal medicine practices to diagnose liver counts the way I would like.
⃝ Frequently ⃝ Occasionally ⃝ Rarely ⃝ Never

**17. How capable do you feel at diagnosing suspected liver disease after detecting an elevated liver count?**

⃝ Very capable ⃝ Rather capable ⃝ Not very capable ⃝ No answer

**18. How would you rate this in general: Are most general practitioners sufficiently capable at diagnosing liver counts, or do you see a need to catch up?**

⃝ Capable enough ⃝ Some need to catch up ⃝ Great need to catch up ⃝ Difficult to say

**19. Do you think it would be helpful if advanced training events were available for general practitioners to diagnose liver counts?**

⃝ Very helpful ⃝ Rather helpful ⃝ Not helpful

**20. In what way would you prefer to attend further training events?**

⃝ Local training ⃝ Online training ⃝ Training DVD ⃝ Training book or similar

*We would like to ask you for some information for statistical purposes. As with the rest of the questionnaire, the information you give will of course be treated in strict confidence and anonymity.*

**You are…** ⃝ Male ⃝ Female ⃝ Diverse

Your **age**:

Your **state**: ⃝ Baden-Württemberg ⃝ Hesse

**Where is your medical practice located?** In a municipality or city with a population of…

⃝ More than 100,000 ⃝ 20,000 to 100,000 ⃝ 5,000 to 20,000 ⃝ Less than 5,000

**Which model** most accurately describes your medical practice?

⃝ Single practice (practice owner is the only doctor) ⃝ Single practice with employed doctors*

⃝ Joint practice* ⃝ Medical centre* ⃝ Other

***How many doctors** work at your practice?

⃝ One doctor ⃝ Two doctors ⃝ Three doctors ⃝ More than three doctors

**How many patients** does your practice treat per quarter?

⃝ 500 to 750 ⃝ 751 to 1,000 ⃝ 1,001 to 1,500 ⃝ More than 1,500

**Thank you for your support!**

Is there anything else you would like to tell us?

Here is space for suggestions, comments, and criticism.
