# Supplementary material for: Evaluation of elevated liver values in primary care - a series of studies on the status quo of care in Germany with special reference to alcoholic liver disease
Source: BMC Prim Care. 2022 May 3;23:104. doi: 10.1186/s12875-022-01714-x (PMC9063320; doi:10.1186/s12875-022-01714-x)
Supplement: Supplementary file 1 — Additional file 1: Appendix 1. Survey of general practitioners [32]. Appendix 2. Survey of gastroenterologists [34]. [file 12875_2022_1714_MOESM1_ESM.zip › questionnaire_specialist survey_translation.docx]

**1. What route do patients with an elevated liver count of unknown aetiology usually take before presenting at your practice?** (Multiple responses as appropriate)

⃝ Referral by a general practitioner ⃝ Referral by another specialist ⃝ Hospital advice

⃝ Direct presentation by the patient ⃝ Other: ⃝ No answer

**2. How many of these patients have clinical symptoms, and how many only have elevated liver values?**

Clinical symptoms: ⃝ Very many ⃝ Rather many ⃝ Average ⃝ Rather few ⃝ Very few ⃝ No answer

Elevated liver count: ⃝ Very many⃝ Rather many ⃝ Average ⃝ Rather few ⃝ Very few ⃝ No answer

**3. Which of the following general and follow-up diagnostic tests for liver diseases does your medical practice provide?**

⃝ Upper abdominal sonography ⃝ Extended laboratory diagnostics ⃝ Elastography ⃝ Liver biopsy ⃝ Gastroscopy

⃝ Ileocolonoscopy ⃝ Other: ⃝ No answer

**4. In general: What do you see as the most frequent indications of incipient liver disease, and what would prompt you to make more in-depth follow-up diagnostics?**

⃝ Long history of alcohol consumption ⃝ Upper abdominal complaints ⃝ Bowel movement and digestion problems

⃝ Tiredness, listlessness ⃝ Recurrent heart problems ⃝ Suspected alcohol abuse

⃝ Persistent diarrhoea ⃝ Chronic pruritus ⃝ Recurrent bladder infection

⃝ Recurrent nosebleeds ⃝ Persistent headache ⃝ Loss of appetite

⃝ Gynaecomastia ⃝ Ascites ⃝ Characteristic skin alterations (spider naevi etc.)

⃝ Carpal tunnel syndrome ⃝ Multiple bruising ⃝ Genital mycosis

⃝ Dupuytren’s contractures ⃝ Changes in bodyweight

Other:

**5. Please give an estimate: How many hepatopathy diagnoses do you think you have performed in the past twelve months?**

⃝ None ⃝ 1 – 10 ⃝ 11 – 20 ⃝ 21 – 30 ⃝ 31 – 40 ⃝ 41 – 50 ⃝ 51 – 60 ⃝ More than 60 ⃝ No answer

**6. How many of your patients have had their first cirrhosis diagnosis in the past twelve months?**

⃝ None ⃝ 1 – 3 ⃝ 4 – 6 ⃝ 7 – 10 ⃝ 11 – 15 ⃝ 16 – 20 ⃝ 21 – 25 ⃝ More than 25 ⃝ No answer

**7. Around how many patients with cirrhosis are currently in treatment at your practice?**

⃝ None ⃝ 1 – 3 ⃝ 4 – 6 ⃝ 7 – 10 ⃝ 11 – 15 ⃝ 16 – 20 ⃝ 21 – 25 ⃝ More than 25 ⃝ No answer

**8. How many of your patients present at your practice with liver disease?**

⃝ Less than 5% ⃝ 6 – 10% ⃝ 10 – 15% ⃝ 16 – 20% ⃝ More than 20% ⃝ No answer

**9. What laboratory findings potentially linked to liver disease do you usually examine in general screening check-ups?**

⃝ Alanine aminotransferase ⃝ Aspartate aminotransferase ⃝ Gamma glutamyltransferase

⃝ Cholinesterase ⃝ Bilirubin ⃝ Albumin ⃝ Alkaline phosphatase

⃝ Ferritin ⃝ Quick/INR ⃝ MCV ⃝ Platelet count

⃝ AMA, AMA/M2 ⃝ Anti-LKM, anti-SLA ⃝ p- and c-ANCA ⃝ Other autoantibodies (ANA etc.)

⃝ Hepatitis B/D ⃝ Hepatitis C ⃝ Hepatitis E ⃝ Immunoglobulins

Other:

**10. If you were forced to decide: What in your opinion would be the three most important indicators in early diagnosis of cirrhosis?**

⃝ Alanine aminotransferase ⃝ Aspartate aminotransferase ⃝ Gamma glutamyltransferase

⃝ Cholinesterase ⃝ Bilirubin ⃝ Albumin ⃝ Alkaline phosphatase

⃝ Ferritin ⃝ Quick/INR ⃝ MCV ⃝ Platelet count

⃝ AMA, AMA/M2 ⃝ Anti-LKM, anti-SLA ⃝ p- and c-ANCA ⃝ Other autoantibodies (ANA etc.)

⃝ Hepatitis B/D ⃝ Hepatitis C ⃝ Hepatitis E ⃝ Immunoglobulins

Other:

**11. How often do you collect liver-associated laboratory findings for control that have already been determined by the general practitioner?**

⃝ Frequently ⃝ Occasionally ⃝ Rarely ⃝ Never ⃝ No answer

**12. How often do you collect additional liver-associated laboratory findings not already determined by the general practitioner?**

⃝ Frequently ⃝ Occasionally ⃝ Rarely ⃝ Never (Please go straight to question 14) ⃝ No answer

**13. Which additional values are collected?**

⃝ Alanine aminotransferase ⃝ Aspartate aminotransferase ⃝ Gamma glutamyltransferase

⃝ Cholinesterase ⃝ Bilirubin ⃝ Albumin ⃝ Alkaline phosphatase

⃝ Ferritin ⃝ Quick/INR ⃝ MCV ⃝ Platelet count

⃝ AMA, AMA/M2 ⃝ Anti-LKM, anti-SLA ⃝ p- and c-ANCA ⃝ Other autoantibodies (ANA etc.)

⃝ Hepatitis B/D ⃝ Hepatitis C ⃝ Hepatitis E ⃝ Immunoglobulins

Other:

**14. Doctors have varying opinions on how to deal with a moderately elevated liver count. Some think it is better to wait after diagnosing a moderately elevated liver count and check them again at a later follow-up. Others prefer direct referral to a specialist physician or clinic. Leaving special cases or unambiguous individual cases aside, what do you think makes more sense?**

⃝ Wait and watch ⃝ Referral to a specialist ⃝ Referral to a specialist liver clinic

⃝ Difficult to say, undecided ⃝ No answer

**15. How long do you think you should wait for the follow-up?**

weeks

**16. Thinking about patients with incipient liver disease you have diagnosed within the last few years: What was the next step of the diagnosis?**

⃝ Patient remained in my consultation for observation or further treatment ⃝ Referral to a specialist liver clinic ⃝ Referral back to the general practitioner for further advice or diagnosis

⃝ Other: ⃝ No answer

**17. How often do you diagnose (incipient) liver disease in a patient that has gone unnoticed or remained undiagnosed in primary care?**

⃝ Frequently ⃝ Occasionally ⃝ Rarely ⃝ Never

**18. In your opinion: How many patients referred to your practice with an elevated liver count ultimately turn out to be non-specific?**

⃝ Very many ⃝ Rather many ⃝ Average ⃝ Rather few ⃝ Very few ⃝ Difficult to say

**19. From your own experience, how would you rate collaboration between resident gastroenterologists and general practitioners in diagnosing elevated liver values of unknown aetiology or cirrhosis?**

⃝ Very good ⃝ Rather good ⃝ Rather poor ⃝ Very poor ⃝ Difficult to say ⃝ No answer

**20. Also from your own experience, how would you rate collaboration between resident gastroenterologists and specialist liver clinics in diagnosing elevated liver values of unknown aetiology or cirrhosis?**

⃝ Very good ⃝ Rather good ⃝ Rather poor ⃝ Very poor ⃝ Difficult to say ⃝ No answer

**21. A variety of challenges may arise when gastroenterologists and general practitioners work together to diagnose and treat cirrhosis. How often have you experienced the following challenges?**

General practitioners are too quick to refer patients with elevated liver values of unknown aetiology to gastroenterologists, leaving gastroenterologists booked out for long periods of time.

⃝ Frequently ⃝ Occasionally ⃝ Rarely ⃝ Never

General practitioners do not adequately inform gastroenterologists about the tests they perform, the results and/or the diagnoses they have made.

⃝ Frequently ⃝ Occasionally ⃝ Rarely ⃝ Never

General practitioners are not always sufficiently aware of elevated liver values with unknown aetiology to notice the onset of liver disease at an early stage.

⃝ Frequently ⃝ Occasionally ⃝ Rarely ⃝ Never

Patients that general practitioners have referred to gastroenterologists for an elevated liver count of unknown aetiology often turn out to be non-specific.

⃝ Frequently ⃝ Occasionally ⃝ Rarely ⃝ Never

General practitioners wait too long before referring patients with an elevated liver count of unknown aetiology to a gastroenterologist.

⃝ Frequently ⃝ Occasionally ⃝ Rarely ⃝ Never

Primary care could do better at initial testing and diagnosis of (incipient) liver disease.

⃝ Frequently ⃝ Occasionally ⃝ Rarely ⃝ Never

I have detected (incipient) liver disease that the general practitioner did not notice or remained unaware of in a patient.

⃝ Frequently ⃝ Occasionally ⃝ Rarely ⃝ Never

General practitioners often fail to follow up on elevated liver values.

⃝ Frequently ⃝ Occasionally ⃝ Rarely ⃝ Never

General practitioners are inconsistent in their approach to analysing liver values; this may include varying liver values recorded depending on the general practitioner, so specialists need to keep adjusting to the preliminary work performed by general practitioners.

⃝ Frequently ⃝ Occasionally ⃝ Rarely ⃝ Never

**22. Cirrhosis is usually diagnosed at an advanced stage because symptoms are relatively non-specific at first. A proposal has been made to establish a structured diagnosis and therapy algorithm giving general practitioners specific instructions on how to deal with elevated liver values and improve care coordination with gastroenterological specialists towards improving early diagnosis.**

**How effective do you think this measure would be in increasing the number of patients diagnosed early?**

⃝ Very effective ⃝ Rather effective ⃝ Not or not very effective ⃝ Don’t know

**23. How capable do you feel at diagnosing suspected liver disease after detecting an elevated liver count?**

⃝ Very capable ⃝ Rather capable ⃝ Not so very capable ⃝ No answer

**24. How capable do you feel about providing treatment and care for patients with cirrhosis?**

⃝ Very capable ⃝ Rather capable ⃝ Not so very capable ⃝ No answer

**25. How would you rate this in general: Are most specialist internists and gastroenterologists sufficiently capable at diagnosing liver disease, or do you see a need to catch up?**

⃝ Sufficiently capable ⃝ Some need to catch up ⃝ Great need to catch up ⃝ Difficult to say

*We would like to ask you for some information for statistical purposes. As with the rest of the questionnaire, the information you give will of course be treated in strict confidence and anonymity.*

**You are…** ⃝ Male ⃝ Female ⃝ Diverse

Your **age**:

Your **federal state**: ⃝ Baden-Württemberg ⃝ Hesse ⃝ Thuringia

**Where is your medical practice located?** In a municipality or city with a population of…

⃝ More than 100,000 ⃝ 20,000 to 100,000 ⃝ 5,000 to 20,000 ⃝ Less than 5,000

**Which model** most accurately describes your medical practice?

⃝ Single practice (practice owner is the only doctor) ⃝ Single practice with employed doctors* ⃝ Joint practice*

⃝ Medical centre*

**You are…**

⃝ Specialist in internal medicine ⃝ Specialist in internal medicine and gastroenterology

⃝ Other:

***How many doctors** are working at your practice?

⃝ One doctor ⃝ Two doctors ⃝ Three doctors ⃝ More than three doctors

**How many patients** does your practice treat per quarter?

⃝ 750 to 1,000 ⃝ 1,000 to 1,500 ⃝ 1,500 to 2,000 ⃝ More than 2,000

**Thank you for your support!**

Is there anything else you would like to tell us?

Here is space for suggestions, comments, and criticism.
